# Use of CRB-65 and quick Sepsis-related Organ Failure Assessment to predict site of care and mortality in pneumonia patients in the emergency department: a retrospective study

**DOI:** 10.1186/s13054-016-1351-0

**Published:** 2016-06-01

**Authors:** Yun-Xia Chen, Jun-Yu Wang, Shu-Bin Guo

**Affiliations:** Emergency Department, Beijing Chao-Yang Hospital Affiliated to Capital Medical University, Chaoyang District, Beijing, 100020 China; No. 8, South Road of Worker’s Stadium, Chaoyang District, Beijing, 100020 China

**Keywords:** qSOFA, CRB-65, Intensive care unit, Emergency department, Prediction

## Abstract

**Background:**

The quick Sepsis-related Organ Failure Assessment (qSOFA) is a new screening system for sepsis that has prognostic performance equal to the full SOFA for patients with suspected infection outside the intensive care unit (ICU). The predictive value of qSOFA for mortality and site of care in patients with pneumonia is not clear. The present study was designed to investigate the predictive performance of qSOFA, CRB-65 (confusion, respiratory rate ≥30/minute, systolic blood pressure <90 mmHg or diastolic blood pressure ≤60 mmHg, age ≥65 years) and CRB (confusion, respiratory rate ≥30/minute, systolic blood pressure <90 mmHg or diastolic blood pressure ≤60 mmHg) for mortality, hospitalisation and ICU admission in patients with pneumonia in the emergency department (ED).

**Methods:**

Retrospective analyses of published data on adult patients with pneumonia presenting between January 2012 and May 2014 were undertaken. The prevalence of 28-day mortality, hospitalisation and ICU admission were compared with regard to qSOFA, CRB and CRB-65 scores. The performance of these three systems for predicting outcomes was compared.

**Results:**

Of 1641 patients, 861 (53 %) were hospitalised (38 % in a general ward, 15 % in the ICU), and the remaining 780 (47 %) were treated as outpatients or were observed in the ED. Within 28 days, 547 (33 %) of 1641 patients died. CRB-65, CRB and qSOFA scores of patients who died, were hospitalised and admitted to the ICU than those who survived and were not hospitalised or admitted to the ICU (*P* < 0.001). AUC values of qSOFA for prediction of 28-day mortality, hospitalisation and ICU admission were similar to those for CRB-65 and CRB. Patients with qSOFA scores of 0, 1, 2 and 3 were associated with, respectively, mortality of 16.3 %, 24.4 %, 48.2 % and 68.4 %; prevalence of hospitalisation of 37.2 %, 47.4 %, 61.6 % and 73.7 %; and prevalence of ICU admission of 9.3 %, 9.1 %, 22.4 % and 45.3 %. Patients with qSOFA scores of 2 and 3 had a significantly higher prevalence of mortality and ICU admission than patients with identical CRB-65 scores.

**Conclusions:**

qSOFA is better than CRB-65 for identification of a high risk of mortality and requirement of ICU admission.

## Background

The definition of sepsis was modified recently, and a novel risk classification system termed the *quick Sepsis-related Organ Failure Assessment* (qSOFA) was recommended for sepsis screening [1]. The qSOFA criteria were respiratory rate ≥22/minute, altered mentation (Glasgow Coma Scale score ≤13 in the original study [2] or <15 in the definitions for sepsis and septic shock set by the Third International Consensus [1]) and systolic blood pressure ≤100 mmHg. In the original study of qSOFA, multivariable logistic regression showed that any two of three criteria offered validity for mortality prediction similar to that of the full Sepsis-related Organ Failure Assessment (SOFA) score for patients with suspected infection outside the intensive care unit (ICU). The AUC of qSOFA for prediction of in-hospital mortality was found to be 0.81.

The qSOFA criteria are very similar to those of CRB-65 (confusion, respiratory rate ≥30/minute, systolic blood pressure <90 mmHg or diastolic blood pressure ≤60 mmHg, age ≥65 years), which have been used widely in patients with community-acquired pneumonia (CAP) [3]. CRB-65 was designed primarily to predict mortality as a simplified system of CURB-65 (confusion, urea >7 mmol/L, respiratory rate ≥30/minute, low systolic [<90 mmHg] or diastolic [≤60 mmHg] blood pressure, age ≥65 years) if data for blood urea are unavailable. In a large, multicentre study involving 388,406 hospitalised patients with CAP, researchers found that CRB-65 could be used to predict death effectively in a three-class pattern, with in-hospital mortality of 2.40 % in class 1 (CRB-65 = 0), 13.43 % in class 2 (CRB-65 = 1 and 2) and 34.39 % in class 3 (CRB-65 = 3 and 4) [4]. In another study, CRB-65 was found to predict mortality similarly to CURB-65 as well as to the Pneumonia Severity Index (PSI), which contains 20 parameters [5]. CRB-65 can be carried out well in low- and moderate-risk patients with CAP, but it has been investigated very rarely in patients with pneumonia at high risk of death. The aim of the present study was to evaluate the prognostic and setting-of-care decision-making performance of qSOFA by comparing it with CRB-65 and CRB in high-risk and unselected patients with pneumonia in the emergency department (ED).

## Methods

### Setting and design of the study

We conducted a retrospective analysis of a previously reported observational clinical study carried out in the ED of Beijing Chao-Yang Hospital (Beijing, China) [6]. The original study was approved by the ethics committee of Beijing Chao-Yang Hospital. Written informed consent was obtained from the patients or their relatives. Care of enrolled participants was provided according to international and local guidelines for pneumonia management in adults [7–9]. Decisions regarding hospitalisation were made by physicians blinded to the study protocol. Patients with high values of CURB-65, a greater number of co-morbidities and a tendency towards deterioration of physical status were hospitalised. Haemodynamically unstable patients who required vasopressors, haemodynamic monitoring and invasive mechanical ventilation and/or continuous renal replacement therapy were admitted to the ICU.

### Study cohort

The researchers in the original study screened consecutive patients with suspected pneumonia who visited the ED between January 2012 and May 2014. Inclusion criteria were age ≥18 years, new infiltrates on chest radiography and two or more symptoms consistent with pneumonia (including cough, dyspnoea, fever, sputum production, breathlessness and/or pleuritic chest pain). Patients with a pulmonary embolism or oedema visualised by computed tomographic angiography of the chest were excluded. Patients with positive test results for HIV or with chronical immunosuppression (including those who had undergone solid organ transplant or splenectomy, had cancer and were undergoing chemotherapy or radiotherapy, and/or were receiving corticosteroids or other immunosuppressive agents), patients with a Do Not Resuscitate order, and those admitted for palliative therapies were also excluded. Enrolled patients included those with CAP or healthcare-associated pneumonia [7].

### Data collection

The researchers in the original study collected general information on enrolled patients upon ED arrival: medical identification, telephone number, demographic characteristics, co-morbidities, vital signs and results of laboratory tests and imaging examinations. CRB-65, CRB and qSOFA scores were calculated for each patient using data obtained from the original study that had been collected upon ED arrival. To assess the illness severity of members of the enrolled cohort, case report forms were reviewed and PSI and Acute Physiology and Chronic Health Evaluation (APACHE) II scores were collected.

### Definition of co-morbidities

Chronic obstructive pulmonary disease (COPD) was defined as a previous diagnosis of COPD. Cardiovascular disease was defined as coronary artery disease (angina or previous myocardial infarction) and/or congestive heart failure (any class of the system set by the New York Heart Association). Stroke was defined as ischaemic and/or haemorrhagic. Tumour was defined as a neoplasm of any type. Renal disease mainly indicated chronic renal failure (including patients undergoing dialysis). Liver disease referred to as cirrhosis of any severity. Diabetes mellitus included insulin-dependent and non-insulin-dependent types.

### Outcome variables

All patients were followed for 28 days through their medical records or by telephone in the primary study, and all-cause mortality at 28 days was the primary outcome. Secondary outcomes were hospitalisation (admission to a general ward or the ICU) and ICU admission.

### Statistical analyses

Data were analysed using SPSS v16.0 software (IBM, Armonk, NY, USA). Data with a normal distribution were expressed as mean ± standard deviation and were analysed by using an independent samples *t* test. Data with a skewed distribution were expressed as medians and quartiles and were analysed by using the Mann-Whitney *U* test. The χ^2^ test was used for comparison of frequencies. To assess the baseline risk of outcomes, the variables of demographics and co-morbidities that were significantly different between patients with opposite outcomes were analysed by binary logistic regression, and the independent predictors were determined. Receiver operating characteristic (ROC) curves were constructed, and the AUC was determined to assess predictive values. For comparison of AUC values, the equation $Z = \kern0.5em \left({\mathrm{A}}_1\kern0.5em -\kern0.5em {\mathrm{A}}_2\right)/\sqrt{\mathrm{S}{\mathrm{E}}_1^2\kern0.5em +\kern0.5em \mathrm{S}{\mathrm{E}}_2^2}$ was used, with test values being Z_0.05_ = 1.96 and Z_0.01_ = 2.58. Prognostic parameters (positive and negative predictive values and positive and negative likelihood ratios) were also calculated. All statistical tests were two-tailed, and *P* < 0.05 was considered significant.

## Results

### Characteristics of the study cohort

As described in the original study, 1769 patients were evaluated in the enrolment period, and 104 patients were excluded because the final diagnosis was not pneumonia. Twenty-four patients were lost to follow-up. We ultimately enrolled 1641 patients with pneumonia, and their baseline characteristics are listed in Table 1. APACHE II scores were calculated for all enrolled patients upon ED arrival, and the median value was 16 (12–21). PSI scores were available for 578 patients, and the mean value was 124 ± 40. Of the whole cohort, 28-day mortality was 33 %, and the prevalence rates of hospitalisation and ICU admission were 52.5 % and 15.0 %, respectively. Median time to ICU admission was 1 (range 1–2) day (day 1 indicated the day of arrival to the ED). Median time of admission to a general ward was 4 (range 2–5) days. The percentage of patients aged ≥65 years was 70.9 % (1163 of 1641).

**Table 1:** Baseline characteristics of the study cohort

Characteristic	Entire cohort	Non-survivors	Survivors	*P* value	Hospitalised	Non-hospitalised	*P* value	ICU admission	Non-ICU admission	*P* value
Number of patients	1641	547	1094		861	780		246	1395	
Age, years	73 (62–79)	75 (66–81)	73 (60–78)	<0.001	74 (65–80)	72 (59–78)	<0.001	75 (68–81)	73 (61–79)	<0.001
Male sex, %	59	58	59	0.48	57	61	0.12	57	59	0.61
Co-morbidities, %										
COPD	34	36	32	0.10	36	31	0.01	43	32	0.001
CVD	28	30	26	0.07	30	26	0.13	30	27	0.33
Stroke	12	14	11	0.08	12	12	0.79	13	12	0.66
Tumour	9	11	8	0.04	10	8	0.16	11	9	0.32
Renal	5	6	4	0.08	6	4	0.22	7	5	0.26
Liver	8	9	8	0.25	8	8	0.62	10	8	0.19
DM	15	13	16	0.13	17	14	0.06	17	15	0.38
Vital signs										
MAP, mmHg	80 (67–93)	70 (54–83)	84 (73–97)	<0.001	77 (60–90)	83 (71–97)	<0.001	67 (50–82)	82 (70–95)	<0.001
Respiratory rate, breaths/minute	27 (24–32)	28 (24–32)	26 (24–32)	0.01	28 (24–32)	26 (24–32)	0.09	30 (24–32)	26 (24–32)	0.003
Temperature, °C	36.8 (36.2–38.2)	36.8 (36.2–38.2)	36.8 (36.2–38.1)	0.83	36.8 (36.2–38.2)	36.8 (36.2–38.1)	0.65	37 (36.2–38.2)	36.8 (36.2–38.1)	0.62
Heart rate, beats/minute	110 (98–126)	110 (99–126)	110 (97–125)	0.20	110 (98–128)	110 (98–124)	0.22	110 (98–126)	110 (98–126)	0.99
Mental confusion, %	28	46	20	<0.001	37	19	<0.001	66	22	<0.001
Laboratory results										
WBC, ×10^9^/L	11.2 (7.7–15.6)	11.0 (7.6–15.1)	11.2 (7.8–15.6)	0.48	11.2 (7.7–15.5)	11.3 (7.7–15.7)	0.75	11.4 (7.6–16)	11.2 (7.7–15.5)	0.71
PCT, ng/ml	1.1 (0.9–3.4)	1.3 (0.9–9.4)	1.1 (0.9–3.4)	<0.001	1.1 (0.9–3.4)	1.1 (0.9–3.4)	0.50	1.2 (0.9–3.7)	1.1 (0.9–3.4)	0.15
PaO_2_, mmHg	75 (59–94)	71 (57–89)	77 (60–96)	<0.001	74 (59–92)	76 (59–96)	0.28	69 (55–86)	77 (60–95)	<0.001
Platelets, ×10^9^/L	184 (135–243)	185 (136–243)	183 (134–244)	0.91	186 (136–243)	183 (134–243)	0.81	188 (142–246)	183 (134–242)	0.73
Creatinine, μmol/L	100 (75–148)	106 (78–170)	98 (74–136)	<0.001	102 (77–157)	98 (73–136)	0.02	134 (98–228)	96 (72–134)	<0.001
Bilirubin, μmol/L	12.3 (8.3–18.6)	13.1 (8.1–19.3)	12.0 (8.1–18.3)	0.04	12.2 (8.2–19)	12.5 (8.3–18.3)	0.94	12.2 (8.2–18.7)	12.3 (8.3–18.5)	0.63
Positive sputum culture	378/1132 (33 %)	143/394 (36 %)	235/738 (32 %)	0.13	209/583 (36 %)	169/549 (31 %)	0.07	73/187 (39 %)	305/945 (32 %)	0.07
Lactate, mmol/L	2.6 (1.0–5.1)	5.4 (3.1–8.1)	1.6 (0.8–3.3)	<0.001	4.4 (2.5–7.0)	1.2 (0.6–2.5)	<0.001	8.5 (6.6–11.7)	2.1 (0.9–4.0)	<0.001
Multi-lobar opacities, %	40	47	37	<0.001	42	38	0.07	53	38	<0.001
PSI	124 ± 40 (*n* = 578)	144 ± 36 (*n* = 199)	113 ± 38 (*n* = 379)	<0.001	137 ± 38 (*n* = 278)	111 ± 38 (*n* = 300)	<0.001	156 ± 37 (*n* = 92)	117 ± 38 (*n* = 486)	<0.001
APACHE II	16 (12–21)	20 (15–26)	15 (11–19)	<0.001	18 (14–23)	15 (11–18)	<0.001	22 (17–28)	16 (11–20)	<0.001
CRB-65	2 (1–2)	2 (1–3)	1 (1–2)	<0.001	2 (1–2)	1 (1–2)	<0.001	2 (2–3)	1 (1–2)	<0.001
CRB	1 (0–1)	1 (1–2)	1 (0–1)	<0.001	1 (0–2)	1 (0–1)	<0.001	1 (1–2)	1 (0–1)	<0.001
qSOFA	1 (1–2)	2 (1–2)	1 (1–1)	<0.001	1 (1–2)	1 (1–2)	<0.001	2 (1–2)	1 (1–2)	<0.001
28-day mortality (%)	33	–	–	–	54	11	<0.001	85	24	<0.001

*Abbreviations: APACHE* Acute Physiology and Chronic Health Evaluation, *COPD* chronic obstructive pulmonary disease, *CRB* confusion, respiratory rate ≥30/minute, systolic blood pressure <90 mmHg or diastolic blood pressure ≤60 mmHg, *CRB-65* confusion, respiratory rate ≥30/minute, systolic blood pressure <90 mmHg or diastolic blood pressure ≤60 mmHg, age ≥65 years, *CVD* cardiovascular disease, *DM* diabetes mellitus, *ICU* intensive care unit, *MAP* mean arterial pressure, *PaO*_*2*_ arterial oxygen pressure, *PCT* procalcitonin, *PSI* Pneumonia Severity Index, *qSOFA* quick Sepsis-related Organ Failure Assessment, *WBC* white blood cells

Skewed distributed data are expressed as medians and quartiles. Normal distributed data are expressed as mean ± standard deviation

### CRB-65, CRB and qSOFA scores and illness severity

CRB-65, CRB, qSOFA, PSI and APACHE II scores were higher in non-survivors than in survivors, in hospitalised than in non-hospitalised patients, and in ICU than in non-ICU patients (*P* < 0.001) (Table 1).

### Baseline risk

Baseline risk variables were age (years), sex and co-morbidities. Variables of age and tumour were different between survivors and non-survivors. Age and COPD were different between patients who were hospitalised or admitted to the ICU and those who were not (Table 1). When analysed by binary logistic regression, age was an independent predictor of all three outcomes. Tumour was not an independent predictor of mortality. For hospitalisation, COPD was an independent predictor without qSOFA, but it was not if it was analysed together with qSOFA. COPD independently predicted ICU admission with or without qSOFA (Table 2). CRB-65 contained age and was equal to CRB plus age, so it was not analysed in binary logistic regression.

**Table 2:** Logistic regression analysis of the baseline risk variables

								95 % CI for OR	
	Outcome	Predictor	β coefficient	SE	Wald statistic	*P* value	OR	5 %	95 %
Without qSOFA	28-day mortality	Age	0.024	0.004	34.321	<0.001	1.025	1.016	1.033
		Tumour	0.322	0.176	3.348	0.067	0.381	0.977	1.950
		Constant	−2.448	0.303	65.404	<0.001	0.086		
	Hospitalisation	Age	0.02	0.004	29.235	<0.001	1.02	1.013	1.027
		COPD	0.232	0.106	4.756	0.029	1.261	1.024	1.552
		Constant	−1.355	0.261	26.962	<0.001	0.258		
	ICU admission	Age	0.024	0.006	17.357	<0.001	1.024	1.013	1.036
		COPD	0.441	0.142	9.65	0.002	1.554	1.177	2.053
		Constant	−3.625	0.431	70.61	<0.001	0.027		
With qSOFA	28-day mortality	Age	0.024	0.004	29.473	<0.001	1.024	1.015	1.033
		Tumour	0.085	0.187	0.207	0.649	1.089	0.754	1.572
		qSOFA	0.939	0.086	119.637	<0.001	2.557	2.161	3.025
		Constant	−3.686	0.34	117.299	<0.001	0.025		
	Hospitalisation	Age	0.019	0.004	25.521	<0.001	1.019	1.012	1.026
		COPD	0.195	0.108	3.264	0.071	1.215	0.984	1.501
		qSOFA	0.522	0.079	43.73	<0.001	1.686	1.444	1.968
		Constant	−1.971	0.282	48.767	<0.001	0.139		
	ICU admission	Age	0.023	0.006	14.252	<0.001	1.023	1.011	1.035
		COPD	0.358	0.147	5.921	0.015	1.431	1.072	1.91
		qSOFA	0.939	0.102	84.913	<0.001	2.557	2.094	3.122
		Constant	−4.881	0.471	107.292	<0.001	0.008		

*qSOFA* quick Sepsis-related Organ Failure Assessment, *COPD* chronic obstructive pulmonary disease, *ICU* intensive care unit

**Fig. 1:**
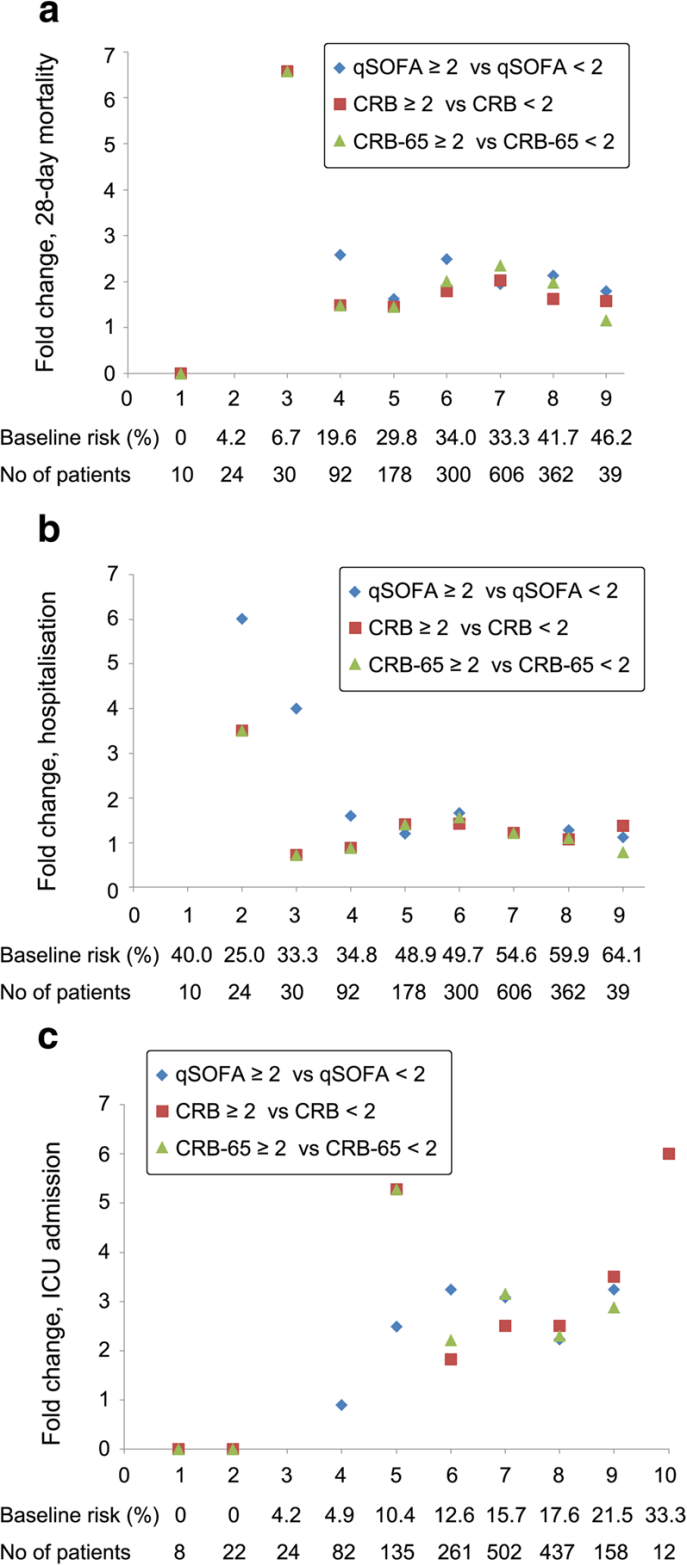
Fold change (ratio) in 28-day mortality, hospitalisation and intensive care unit (ICU) admission of patients with scores ≥2 vs. <2. **a** Twenty-eight-day mortality. **b** Hospitalisation. **c** ICU admission. Twenty-eight-day mortality of patients in baseline risk class 1 was zero. Twenty-eight-day mortality of patients with quick Sepsis-related Organ Failure Assessment (qSOFA) score <2 was zero in baseline risk classes 2 and 3. Twenty-eight-day mortality of patients with CRB/CRB-65 scores <2 was zero in baseline risk class 2. Prevalence of hospitalisation of patients in baseline risk class 1 was zero when the score was ≥2 for qSOFA, CRB and CRB-65. Prevalence of ICU admission was zero in patients in baseline risk classes 1 and 2. Prevalence of ICU admission was zero in patients with qSOFA score ≥2 in baseline risk class 3. Prevalence of ICU admission was zero in patients with qSOFA score <2 in baseline risk class 10. Prevalence of ICU admission was zero in patients with CRB/CRB-65 scores ≥2 in baseline risk classes 3 and 4. *CRB* confusion, respiratory rate ≥30/minute, systolic blood pressure <90 mmHg or diastolic blood pressure ≤60 mmHg, *CRB-65* confusion, respiratory rate ≥30/minute, systolic blood pressure <90 mmHg or diastolic blood pressure ≤60 mmHg, age ≥65 years

The baseline risk model for mortality and hospitalisation contained only age. Patients were divided into nine baseline risk classes according to increasing decades (class 1 age 18–19 years, class 2 age 20–29 years, and so forth). The baseline risk model for ICU admission contained age and COPD. A patient with COPD was defined as 1 point. For example, a 45-year-old patient with COPD was defined as class 5. The baseline risks for mortality, hospitalisation and ICU admission are shown in Fig. 1. Except for hospitalisation, the prevalence of outcomes increased directly with baseline risk classes. The prevalence of hospitalisation for baseline risk class 1 was 40 %, and it was higher than that for classes 2, 3 and 4. There was a ten-fold variation in 28-day mortality across classes of baseline risk from 0 % to 46.2 %. For ICU admission, the variation across baseline risk classes ranged from 0 % to 33.3 %. Hospitalisation varied from 25 % to 64.1 % according to baseline risk classes (Fig. 1).

### Predictive performance of the baseline risk model, CRB-65, CRB and qSOFA

**Fig. 2:**
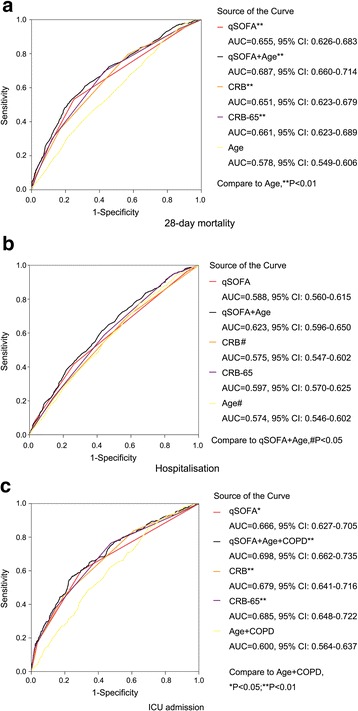
Receiver operating characteristic curves of the baseline risk model, CRB-65, CRB and qSOFA for predicting outcomes. **a** Twenty-eight-day mortality. **b** Hospitalisation. **c** ICU admission. Baseline model: age for 28-day mortality and hospitalisation; age + COPD for ICU admission. *COPD* chronic obstructive pulmonary disease, *ICU* intensive care unit, *qSOFA* quick sepsis-related organ failure assessment, *CRB* confusion, respiratory rate ≥30/minute, systolic blood pressure <90 mmHg or diastolic blood pressure ≤60 mmHg, *CRB-65* confusion, respiratory rate ≥30/minute, systolic blood pressure <90 mmHg or diastolic blood pressure ≤60 mmHg, age ≥65 years

ROC curves for the baseline risk model (age for mortality and hospitalisation, age + COPD for ICU admission), CRB-65, CRB and qSOFA are shown in Fig. 2. AUC values of the baseline risk model ranged from 0.57 to 0.60 for prediction of the three outcomes. AUC values of the baseline risk model in prediction of mortality and ICU admission were much lower than those for CRB, CRB-65 and qSOFA (*P* < 0.05). For prediction of hospitalisation, age achieved a similar AUC value for the three scoring systems, and showed a significant improvement by combination with qSOFA (*P* < 0.05). AUC values for CRB, CRB-65 and qSOFA for prediction of the three outcomes varied from 0.57 to 0.68 and were not significantly different from each other (*P* > 0.05). Combination of the baseline risk model with qSOFA did not improve the performance of qSOFA significantly for prediction of all three outcomes. Cut-off values are listed in Table 3.

**Table 3:** Predictive performance of CRB-65, CRB and qSOFA

										95 % CI	
Outcomes	Predictors	Cut-off value	Sensitivity	Specificity	PPV	NPV	LR^+^	LR^−^	OR	5 %	95 %
Mortality	CRB-65	≤1	70 %	57 %	45 %	79 %	1.6	0.5	3.059	2.459	3.804
	CRB-65	2	30 %	88 %	55 %	72 %	2.5	0.8	3.120	2.409	4.041
	CRB-65	≥3	7 %	98 %	60 %	68 %	3.5	1.0	3.141	1.854	5.321
	CRB	≤1	36 %	81 %	51 %	70 %	1.9	0.8	2.442	1.927	3.094
	CRB	2	9 %	97 %	62 %	66 %	3.0	0.9	3.201	1.994	5.139
	qSOFA	≤1	53 %	75 %	52 %	76 %	2.1	0.6	3.418	2.752	4.246
	qSOFA	2	12 %	97 %	68 %	69 %	4.0	0.9	4.783	3.063	7.469
Hospitalisation	CRB-65	≤1	59 %	56 %	60 %	55 %	1.3	0.7	1.807	1.485	2.198
	CRB-65	2	22 %	86 %	63 %	50 %	1.6	0.9	1.715	1.322	2.224
	CRB-65	≥3	5 %	97 %	67 %	48 %	1.7	1.0	1.851	1.073	3.195
	CRB	≤1	27 %	81 %	62 %	50 %	1.4	0.9	1.630	1.290	2.060
	CRB	2	6 %	97 %	69 %	48 %	2.0	1.0	2.066	1.263	3.381
	qSOFA	≤1	42 %	74 %	64 %	53 %	1.6	0.8	2.006	1.627	2.473
	qSOFA	2	8 %	97 %	74 %	49 %	2.7	1.0	2.673	1.675	4.265
ICU admission	CRB-65	≤1	76 %	53 %	22 %	93 %	1.6	0.5	3.589	2.626	4.906
	CRB-65	2	38 %	86 %	32 %	89 %	2.7	0.7	3.611	2.681	4.864
	CRB-65	≥3	14 %	98 %	57 %	87 %	7.0	0.9	8.444	4.967	14.357
	CRB	≤1	45 %	81 %	29 %	89 %	2.4	0.7	3.426	2.579	4.552
	CRB	2	15 %	97 %	49 %	87 %	5.0	0.9	6.352	3.970	10.163
	qSOFA	≤1	60 %	70 %	26 %	91 %	2.0	0.6	3.554	2.687	4.702
	qSOFA	2	18 %	96 %	45 %	87 %	4.5	0.9	5.471	3.558	8.411

*Abbreviations: qSOFA* quick Sepsis-related Organ Failure Assessment, *PPV* positive predictive value, *NPV* negative predictive value, *LR* ^*+*^ positive likelihood ratio, *LR* ^*−*^ negative likelihood ratio, *ICU* intensive care unit, *CRB* confusion, respiratory rate ≥30/minute, systolic blood pressure <90 mmHg or diastolic blood pressure ≤60 mmHg, *CRB-65* confusion, respiratory rate ≥30/minute, systolic blood pressure <90 mmHg or diastolic blood pressure ≤60 mmHg, age ≥65 years

**Fig. 3:**
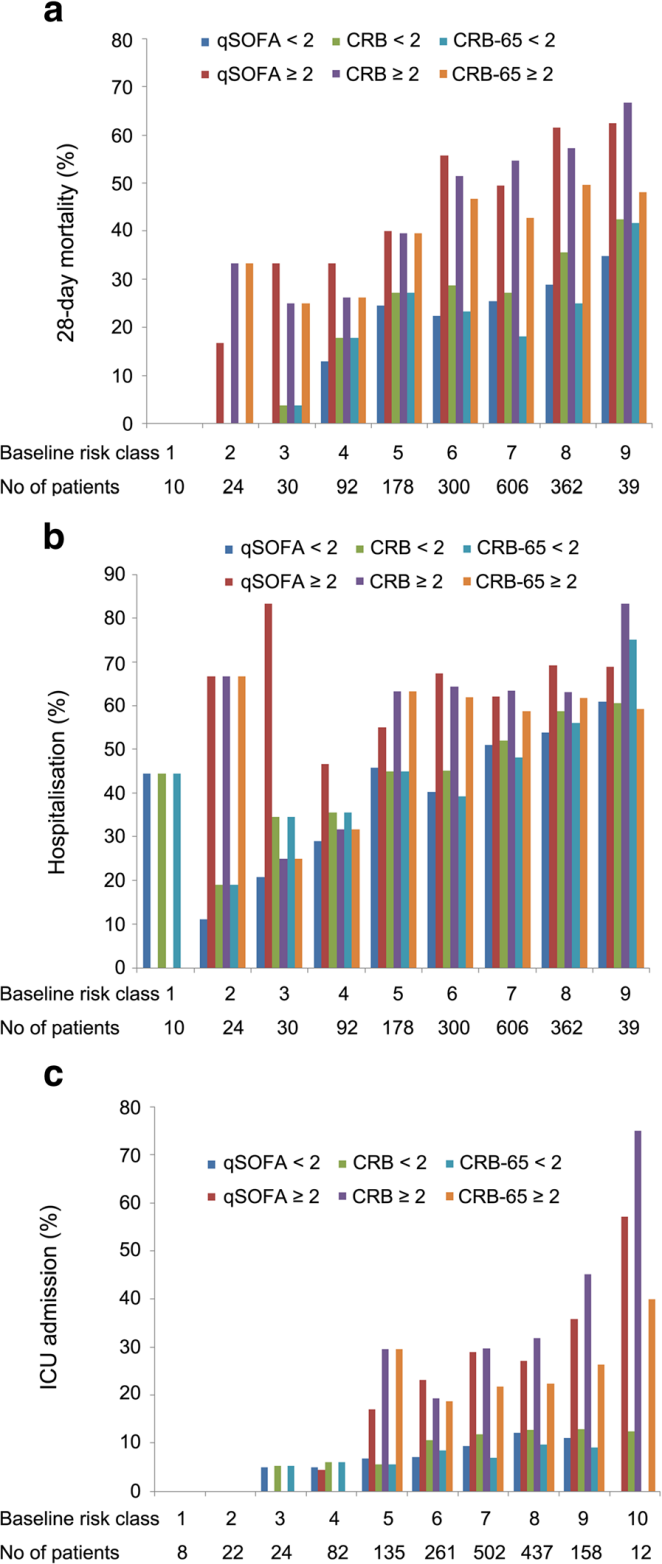
Prevalence of outcomes according to CRB-65, CRB and qSOFA scores. **a** Twenty-eight-day mortality. **b** Hospitalisation. **c** ICU admission. *qSOFA* quick Sepsis-related Organ Failure Assessment, *ICU* intensive care unit, *CRB* confusion, respiratory rate ≥30/minute, systolic blood pressure <90 mmHg or diastolic blood pressure ≤60 mmHg, *CRB-65* confusion, respiratory rate ≥30/minute, systolic blood pressure <90 mmHg or diastolic blood pressure ≤60 mmHg, age ≥65 years

The additional risk-predictive performance of CRB, CRB-65 and qSOFA above that of the baseline risk model was also investigated. Cut-off values were defined as a score ≥2 in all three systems because the threshold achieved specificities of 86–97 % for prediction of all three outcomes (Table 3). The prevalence of the three outcomes is shown in Fig. 3. Fold changes are shown in Fig. 1. Over baseline risk classes, patients with a qSOFA scores ≥2 vs. <2 had a two- to three-fold increase in 28-day mortality. Within baseline risk classes, the fold change of mortality between patients with CRB/CRB-65 scores ≥2 and <2 was less than that observed for qSOFA. Compared with patients with qSOFA scores <2, those with qSOFA scores ≥2 had a one- to six-fold increase in the prevalence of hospitalisation. In baseline risk classes 2–4, the fold change of hospitalisation was two- to six-fold for qSOFA and was more obvious than that for CRB/CRB-65 (one- to four-fold). In baseline risk class ≥5, fold changes of the three systems were similar. Patients with qSOFA scores ≥2 had a one- to three-fold increase in the prevalence of ICU admission compared with patients with qSOFA scores <2.

### Prevalence of outcomes according to CRB-65, CRB and qSOFA scores

The prevalence of outcomes according to CRB-65, CRB and qSOFA scores are shown in Table 4. Twenty-eight-day mortality and ICU admission of patients with qSOFA scores of 2 and 3 were much higher than those of patients with the same CRB-65 scores (*P* < 0.01). The prevalence of all three outcomes of patients with a CRB score of 1 was much higher than that for patients with a qSOFA score of 1.

**Table 4:** prevalence of outcomes according to qSOFA, CRB-65 and CRB scores

	Score				
	0	1	2	3	4
Number of patients					
qSOFA	86	991	469	95	–
CRB-65	165	626	556	234	60
CRB	582	678	304	77	–
28-day mortality, *n* (%)					
qSOFA	14 (16.3)	242 (24.4)^a^	226 (48.2)^a^	65 (68.4)	–
CRB-65	28 (17.0)	138 (22.0) ^a^	218 (39.2)^a,b^	127 (54.3)^b^	36 (60.0)
CRB	111 (19.1)^a^	240 (35.4)^a,b^	148 (48.7)^c^	48 (62.3)	–
Hospitalization, *n* (%)					
qSOFA	32 (37.2)	470 (47.4)^a^	289 (61.6)^c^	70 (73.7)	–
CRB-65	46 (27.9)^a^	309 (49.4)^a^	320 (57.6)	146 (62.4)	40 (66.7)
CRB	257 (44.2)^a^	369 (54.4)^b^	182 (59.9)	53 (68.8)	–
ICU admission, *n* (%)					
qSOFA	8 (9.3)	90 (9.1)^a^	105 (22.4)^a^	43 (45.3)	–
CRB-65	13 (7.9)	45 (7.2)^a^	95 (17.1)^a,b^	59 (25.2)^a,b^	34 (56.7)
CRB	40 (6.9)^a^	95 (14.0)^a,b^	73 (24.0)^a^	38 (49.4)	–

*Abbreviations: qSOFA* quick sepsis-related organ failure assessment, *ICU* intensive care unit, *CRB* confusion, respiratory rate ≥30/minute, systolic blood pressure <90 mmHg or diastolic blood pressure ≤60 mmHg, *CRB-65* confusion, respiratory rate ≥30/minute, systolic blood pressure <90 mmHg or diastolic blood pressure ≤60 mmHg, age ≥65 years

^a^ *P* < 0.01 compared with the next score value in each score system,

^b^ *P* < 0.01 compared with the same score value of qSOFA

^c^ *P* < 0.05 compared with the next score value in each score system

## Discussion

The present study revealed that the ability of qSOFA to identify patients with pneumonia in the ED at high risk of death and requirement of ICU admission was better than that for CRB-65. CRB and qSOFA contain three identical vital signs: respiratory rate, mentation and blood pressure. The criteria thresholds of respiratory rate and blood pressure were stricter for CRB than for qSOFA. The method of assessment for altered mentation was simpler for qSOFA than for CRB. Therefore, CRB was expected to be more accurate than qSOFA for predicting outcomes. However, in the present study, the ROC curves of CRB-65, CRB and qSOFA were similar with regard to prediction of all three outcomes. AUC values of CRB-65 and CRB were higher than that of qSOFA, but the differences were not significant. Age ≥65 years was included in the CRB-65, but it did not provide additional predictive performance in the present study. The reduced predictive value of age could have been due to 70.9 % of the cohort being aged ≥65 years. Despite the differences in criteria threshold and assessment method of altered mentation, the novel and simpler qSOFA achieved general performance for prediction of mortality and site of care equal to that of CRB-65, which has been verified to be effective and has been used widely for several years [4, 10–12].

AUC of qSOFA in the present study was much lower than that in the original study for predicting mortality in patients suspected of having an infection outside the ICU (0.655 vs. 0.81) and was similar to the AUC of ICU encounters (0.655 vs. 0.66) [2]. One reason may be that illness severity in the cohort of the present study was close to that for ICU encounters. Age was an independent baseline risk variable, but it did not improve the prognostic performance of qSOFA upon its combination with qSOFA. Similarly to the original study, qSOFA was of additional prognostic value above baseline risk. The fold changes of mortality between qSOFA score ≥2 and qSOFA score <2 within baseline risk class were more significant in low baseline risk classes and higher than that for CRB/CRB-65.

In the original study of CRB-65, the AUC was not provided [3]. In other studies involving CRB-65, the AUC varied from 0.69 to 0.82 for prediction of mortality in patients with CAP [5, 10–13]. For CRB-65, the AUC for mortality was 0.661 in the present study, which is lower than the AUC values in those studies. The larger number of critically ill patients may have been the main reason for the low AUC values of CRB-65 and qSOFA in the present study. This hypothesis is supported by the higher illness severity scores (PSI 124 ± 40, APACHE II 16 [12–21]) and mortality (33 %) observed in the present study. In critically ill patients, the effectiveness of using simple systems to gauge severity was reduced, and additional variables were needed. The original study of qSOFA found that the prognostic value of qSOFA was better for patients outside the ICU (AUC 0.81) than for patients within the ICU (AUC 0.66), whereas SOFA (AUC 0.74) was more effective than qSOFA for predicting mortality in ICU populations [2]. Use of CRB-65 for predicting mortality has been identified in a large multicentre study that enrolled 388,406 hospitalised patients with CAP [4]. In that study, overall in-hospital mortality was 14.1 %, and CRB-65 predicted death in a three-class pattern, with in-hospital mortality of 2.40 % in class 1 (CRB-65 = 0), 13.43 % in class 2 (CRB-65 = 1 and 2) and 34.39 % in class 3 (CRB-65 = 3 and 4). AUC was not provided in that study, but the results confirmed the good prognostic ability of CRB-65 in a cohort of patients with moderate risk CAP. In the present study, qSOFA showed better performance for predicting mortality than CRB-65 if scores were ≥2.

AUC values of CRB-65, CRB and qSOFA for hospitalisation were <0.6, which indicated poor predictive value. We attributed this result to the relatively larger number of critically ill patients for whom additional variables of risk stratification needed to be applied. The advantage of qSOFA was that it provided an additional stronger value above baseline risk than CRB/CRB-65 for predicting hospitalisation in patients younger than 50 years of age.

One study showed that the prevalence of ICU admission increased directly with the risk class of CRB-65 in a cohort of hospitalised patients with CAP in Hong Kong [5]. However, the prevalence of ICU admission (4.0 %) was significantly less than that observed in the present study. In another study, 10.2 % of patients required invasive ventilation and/or inotropic support (the main criteria for ICU admission), and CRB-65 achieved an AUC of 0.77 for predicting the need for mechanical ventilation and/or inotropic support [13]. In the present study, CRB-65, CRB and qSOFA could be used to predict ICU admission. The difference in the prevalence of ICU admission between score values was more significant in qSOFA than in CRB-65. Therefore, qSOFA was more effective than CRB-65 for identifying high-risk patients who needed ICU treatment.

### Limitations

The first limitation is that we conducted a single-centre study. Hospitalisation and decisions made regarding ICU admission were based on institutional or departmental guidelines, which played a part in site-of-care decisions. Multicentre prospective studies may reduce this influence.

The second limitation is that the high mortality in the study cohort may limit the generalisability of our results to a certain extent. The high mortality was due to seven main reasons. The first reason was the advanced age of the study cohort. Second, patients with pneumonia with co-morbidities tended to also develop multiple organ dysfunction syndrome. Third, as a large tertiary teaching hospital, our institution received numerous patients with severe pneumonia transferred from smaller hospitals and primary healthcare institutions. Before being transferred to our ED, these patients were hospitalised, treated with more than one antibiotic and perhaps intubated and ventilated mechanically, but these interventions were not effective. Fourth, few low-risk patients with pneumonia were enrolled in the ED, because almost all patients at high risk of death are sent to an ED for initial assessment in China; in contrast, many patients with less severe illnesses visit specialist clinics directly and are not evaluated in an ED, and this situation is more significant in a large academic tertiary hospital. Fifth, patients admitted to the ICU had developed multiple organ dysfunction, were intubated and ventilated mechanically, and needed dynamic monitoring and support or continuous renal replacement therapy. Sixth, bedridden patients with repeated pneumonia and patients resident in nursing homes were included. Seventh, though treatment was administered according to guidelines, assessment of its effectiveness was not included in the present study. These seven factors tended to result in a higher prevalence of mortality. In a CAP cohort that excluded the patients mentioned above, mortality was relatively low. The Genetic and Inflammatory Markers of Sepsis Study excluded patients who were transferred from other hospitals, were discharged from a hospital within the previous 10 days, had had an episode of pneumonia within the previous 30 days, had undergone chronic mechanical ventilation, had cystic fibrosis or active pulmonary tuberculosis, were admitted for palliative care, were enrolled in a previous study, were incarcerated, and were pregnant. Also, overall 90-day mortality was 10.4 %, which was much lower than that observed in our study [14].

The third limitation is that most patients requiring invasive mechanical ventilation, vasopressors or continuous renal replacement therapy were admitted to an ICU in our study, but an accurate record of life support was not available. Though our study had these three main limitations, we investigated the potential prognostic and triage value of qSOFA in a relatively high-risk population that was different from usual CAP cohorts.

## Conclusions

CRB-65 and qSOFA were of equal general value for predicting mortality and assessment of the level of care in high-risk patients with pneumonia in the ED. qSOFA was more effective than CRB-65 in distinguishing a high risk of mortality and requirement for ICU admission.

## Key messages

- qSOFA criteria were respiratory rate ≥22 breaths/minute, altered mentation (Glasgow Coma Scale score ≤13 in the original study or <15 in the definitions for sepsis and septic shock set by the Third International Consensus) and systolic blood pressure ≤100 mmHg.
- In the present study, patients with qSOFA scores of 0, 1, 2 and 3 had, respectively, mortality of 16.3 %, 24.4 %, 48.2 % and 68.4 %; prevalence of hospitalisation of 37.2 %, 47.4 %, 61.6 % and 73.7 %; and prevalence of ICU admission of 9.3 %, 9.1 %, 22.4 % and 45.3 %.
- qSOFA provided additional value above baseline risk in predicting mortality and requirement for ICU admission.
- AUC values of qSOFA for prediction of mortality, hospitalisation and ICU admission were similar to those of CRB-65 in patients with pneumonia in the ED.
- qSOFA provided better performance than CRB-65 for distinguishing a high risk of mortality and requirement for ICU admission in patients with pneumonia in the ED.

## Abbreviations

APACHE, Acute Physiology and Chronic Health Evaluation; CAP, community-acquired pneumonia; COPD, chronic obstructive pulmonary disease; CRB, confusion, respiratory rate ≥30/minute, systolic blood pressure <90 mmHg or diastolic blood pressure ≤60 mmHg; CRB-65, confusion, respiratory rate ≥30/minute, systolic blood pressure <90 mmHg or diastolic blood pressure ≤60 mmHg, age ≥65 years; CURB-65, confusion, urea >7 mmol/L, respiratory rate ≥30/minute, low systolic (<90 mm Hg) or diastolic (≤60 mmHg) blood pressure), age ≥65 years; CVD, cardiovascular disease; DM, diabetes mellitus; ED, emergency department; ICU, intensive care unit; LR, likelihood ratio; MAP, mean arterial pressure; NPV, negative predictive value; PaO_2_, arterial oxygen pressure; PCT, procalcitonin; PPV, positive predictive value; PSI, Pneumonia Severity Index; qSOFA, quick Sepsis-related Organ Failure Assessment; ROC, receiver operating characteristic; SOFA, full Sepsis-related Organ Failure Assessment; WBC, white blood cells

## References

[1] Singer M, Deutschman CS, Seymour CW, Shankar-Hari M, Annane D, Bauer M (2016). The Third International Consensus Definitions for Sepsis and Septic Shock (Sepsis-3). JAMA.

[2] Seymour CW, Liu VX, Iwashyna TJ, Brunkhorst FM, Rea TD, Scherag A (2016). Assessment of clinical criteria for sepsis: for the Third International Consensus Definitions for Sepsis and Septic Shock (Sepsis-3). JAMA.

[3] Lim WS, van der Eerden MM, Laing R, Boersma WG, Karalus N, Town GI (2003). Defining community acquired pneumonia severity on presentation to hospital: an international derivation and validation study. Thorax.

[4] Ewig S, Birkner N, Strauss R, Schaefer E, Pauletzki J, Bischoff H (2009). New perspectives on community-acquired pneumonia in 388 406 patients: results from a nationwide mandatory performance measurement programme in healthcare quality. Thorax.

[5] Man SY, Lee N, Ip M, Antonio GE, Chau SS, Mak P (2007). Prospective comparison of three predictive rules for assessing severity of community-acquired pneumonia in Hong Kong. Thorax.

[6] Chen YX, Li CS (2015). Lactate on emergency department arrival as a predictor of mortality and site-of-care in pneumonia patients: a cohort study. Thorax.

[7] American Thoracic Society; Infectious Diseases Society of America (2005). Guidelines for the management of adults with hospital-acquired, ventilator-associated, and healthcare-associated pneumonia. Am J Respir Crit Care Med.

[8] Lim WS, Baudouin SV, George RC, Hill AT, Jamieson C, Le Jeune I (2009). BTS guidelines for the management of community acquired pneumonia in adults: update 2009. Thorax.

[9] Mandell LA, Wunderink RG, Anzueto A, Bartlett JG, Campbell GD, Dean NC (2007). Infectious Diseases Society of America/American Thoracic Society consensus guidelines on the management of community-acquired pneumonia in adults. Clin Infect Dis..

[10] Arnold FW, Brock GN, Peyrani P, Rodríguez EL, Díaz AA, Rossi P (2010). Predictive accuracy of the pneumonia severity index vs CRB-65 for time to clinical stability: results from the Community-Acquired Pneumonia Organization (CAPO) International Cohort Study. Respir Med.

[11] Dwyer R, Hedlund J, Henriques-Normark B, Kalin M (2014). Improvement of CRB-65 as a prognostic tool in adult patients with community-acquired pneumonia. BMJ Open Respir Res.

[12] Kolditz M, Höffken G, Martus P, Rohde G, Schütte H, Bals R (2012). Serum cortisol predicts death and critical disease independently of CRB-65 score in community-acquired pneumonia: a prospective observational cohort study. BMC Infect Dis..

[13] Chalmers JD, Singanayagam A, Hill AT (2008). Systolic blood pressure is superior to other haemodynamic predictors of outcome in community acquired pneumonia. Thorax.

[14] Kellum JA, Kong L, Fink MP, Weissfeld LA, Yealy DM, Pinsky MR (2007). Understanding the inflammatory cytokine response in pneumonia and sepsis: results of the Genetic and Inflammatory Markers of Sepsis (GenIMS) Study. Arch Intern Med.

